# Phytolith Production and Morphotypes in Modern Plants on the Tibetan Plateau

**DOI:** 10.3389/fpls.2022.950322

**Published:** 2022-07-11

**Authors:** Yong Ge, Yingshuai Jin, Xiaoling Zhang

**Affiliations:** ^1^Department of Archaeology and Anthropology, University of Chinese Academy of Sciences, Beijing, China; ^2^Key Laboratory of Vertebrate Evolution and Human Origins, Institute of Vertebrate Paleontology and Paleoanthropology, Chinese Academy of Sciences, Beijing, China; ^3^Center for Excellence in Life and Paleoenvironment, Chinese Academy of Sciences, Beijing, China

**Keywords:** phytolith production, phytolith morphology, Poaceae, Cyperaceae, Tibetan Plateau

## Abstract

Tibetan Plateau is the “third pole” of Earth and significantly influences the world’s ecosystems. However, limited work on phytolith analysis has been done due to its harsh environment, and no study on phytolith production and morphotypes in modern plants on the Tibetan Plateau has been carried out yet. In this study, we investigated 73 modern plant samples collected on the Tibetan Plateau to study phytolith production and morphology. The results showed that the major phytolith producers are Poaceae and Cyperaceae plants, the production of phytolith is higher than 0.4 million grains/g in most samples. We found one new morphotype, BILOBATE SADDLE, which could be the diagnostic type for Tribe Stipeae and phytoliths morphotypes might indicate different hydrological conditions on the Tibetan Plateau. Our findings add new information about phytoliths on the Tibetan Plateau and will aid the future phytolith analysis in this region.

## Introduction

The Tibet Plateau (TP) is well known as the “Earth’s Third Pole,” with an average altitude of 4,000 m (Zheng and Yao, 2004). The TP has a unique but vulnerable ecosystem due to the anoxic and strong ultraviolet radiation environment, which is highly sensitive to climate change and human activities (Yao and Zhu, 2015). Therefore, it is crucial to understand the past environmental changes for policy-making and address future climate changes (Chen D.L. et al., 2015). The TP also played an important role in understanding the dispersal of modern humans in Asia (Bae et al., 2017). Meanwhile, the time humans appeared on the TP (Zhang et al., 2018; Chen et al., 2019) and their permanent occupation of the TP (Chen F.H. et al., 2015) are fascinating topics in archeology. However, much more details of how and when humans conquered the TP remain to be studied.

In TP, fossil pollen assemblages and charred seeds are most used as indicators for the past environment (Tang et al., 2021) and agricultural activities (Gao et al., 2020; Ren et al., 2020; Wang et al., 2020), respectively. However, less attention has been paid to phytolith analysis (Chen et al., 2008). Phytolithsr are micro silica bodies originating from plant cells (Piperno, 1988), which are of high taxonomical value and have been applied in reconstructing the paleoenvironment (Barboni et al., 2010; Gu et al., 2012; Stromberg et al., 2013; Zhang et al., 2020; Li et al., 2021) and prehistory agricultural activities (Piperno et al., 2009; Deng et al., 2017; Wang et al., 2017; Zhang et al., 2017; Dunseth et al., 2019; Huan et al., 2022) in many regions. In contrast with pollen and charred seeds, the *in situ* deposition of phytoliths could provide more local information (Rovner, 1971) and phytoliths are more durable in fire pits or environments where organic matter is hard to preserve (Piperno, 2006). Previous studies implied that regional modern phytoliths references are crucial for the better interpretation of sedimental phytoliths assemblages (Fredlund and Tieszen, 1997; Prebble et al., 2002; Lu and Liu, 2005; Lu et al., 2006; Liu et al., 2018). Thus, phytolith analysis could be a promising tool for reconstructing TP’s paleoenvironment and past agricultural activities.

The primary vegetation type on the TP is alpine grassland and alpine meadow, and the dominant species are mainly from Poaceae and Fabaceae (Tsering et al., 2021), which are typical phytoliths producers and non-producers. The most cultivated crops on the TP are naked barley (*Hordeum vulgare* var. *nudum*), which is also a phytolith producer. However, no study on modern plant phytoliths has been published yet, which resulted in a lack of knowledge on diagnostic phytolith types and the relationship between phytoliths and environmental factors on the TP. Thus, the study on modern plants could provide insight into the phytolith assemblages and further aid the studies on reconstructing the regional paleoenvironment and past agricultural activities on the TP.

## Materials and Methods

**FIGURE 1:**
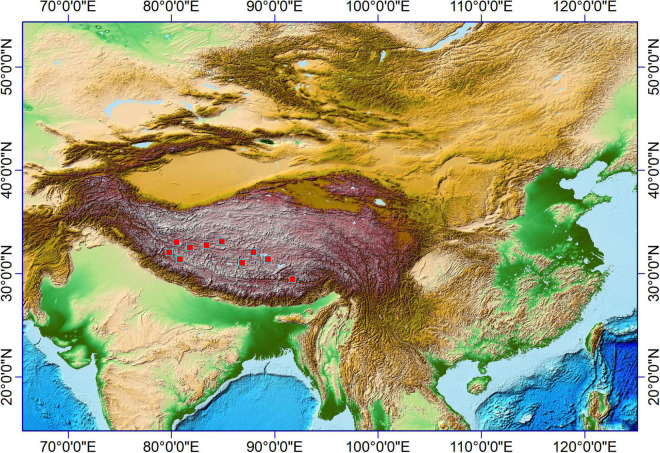
A map showed the Tibet Plateau and the surrounding regions. The red box shows the sampling sites.

The samples for this study mainly were collected on the northern high plateau of Tibet (above 4,000 m) in July and August 2018, where the vegetation type is the alpine meadow (Tsering et al., 2021). The growing season is from June to August in the northern high plateau of Tibet, when the monthly temperature and precipitation could be above 10°C and 20 mm, respectively, (Hu et al., 2022); the sampling sites in this region (Figure 1) shared the same climate. Plant samples collected in this study were fully ripened and could be found with seeds (if not eaten by animals). However, most plant samples were no higher than 20 cm due to the grazing in these regions, and inflorescences were mostly eaten by animals. Thus, the identification of plant samples was limited to genera level and sometimes to species level (if applicable). A total of 73 plant specimens were involved in this study and ultrasonically cleaned with distilled water to clean the surface of collected aerial parts; all samples were dried in a drier and then weighted before phytolith extraction.

Phytolith extraction followed the previously published wet oxidation method (Ge et al., 2020b). Additional polypodium tablet (ca. 27560 grains per tablet) is added to estimate the phytolith yield in each specimen (one tablet for one specimen). Phytolith count in each sample was above 300 grains, except for *Salix bangongensis*, in which we observed only one phytolith in two slides. As we knew that some taxa might not produce phytoliths, 21 plant specimens from Fabaceae, Asteraceae, Polygonaceae, and Primulaceae were identified at the family level, and after phytolith extraction, they were examined to be non-phytolith producers. Thus, only 52 samples (Table 1) were applied for further analysis. The identification and imaging of phytoliths were under 400× with a Leica DM750 microscope. Phytolith nomenclature follows the ICPN 2.0 rules (Neumann et al., 2019) and a previous study (Ge et al., 2020a).

**TABLE 1:** Sampling information and phytolith production of studied specimens.

Sample number	Original code	Specimen (Latin name)	Phytolith yield (grains/g)	N	E	Elevation (m)	Description	Date
Plant 44	ZW-ALGJ-1	*Elymus* sp.	895952	32°22′	80°37′	4770	Open area near the main road	2018/7/23
Plant 45	ZW-ALGJ-2	*Stipa* sp.	5306509	32°22′	80°37′	4770	Open area near the main road	2018/7/23
Plant 25	ZW-DZH-1	*Poa* sp.	7695325	32°26′	80°2′	4264	The dry lake basin of the Dingzhong Lake	2018/7/16
Plant 26	ZW-DZH-2	*Elymus atratus*	12371378	32°26′	80°2′	4264	The dry lake basin of the Dingzhong Lake	2018/7/16
Plant 27	ZW-DZH-4	*Elymus dahuricus*	6021261	32°26′	80°2′	4296	The dry lake basin of the Dingzhong Lake	2018/7/16
Plant 28	ZW-DZH-6	*Elymus nutans*	3819272	32°26′	80°2′	4264	Mountain slope near the Dingzhong Lake	2018/7/16
Plant 47	ZW-GJ-1	*Hordeum vulgare* var. *nudum*_straw	1091662	32°23′	81°1′	4509	In the garden of the hotel, the straw	2018/8/1
Plant 48	ZW-GJ-2	*Hordeum vulgare* var. *nudum*_leaf	3924019	32°23′	81°1′	4509	In the garden of the hotel, the leave	2018/8/1
Plant 49	ZW-GJ-3	*Hordeum vulgare* var. *nudum* _inflorescence	1576348	32°23′	81°1′	4509	In the garden of the hotel, the inflorescence	2018/8/1
Plant 18	ZW-GZ-1	*Kobresia robusta*	1806164	31°52′	85°58′	4851	Near a small lake next to the main road	2018/7/14
Plant 19	ZW-GZ-2	*Pennisetum alopecuroides*	2435948	31°52′	85°58′	4851	Near a small lake next to the main road	2018/7/14
Plant 20	ZW-GZ-3	*Stipa* sp.	3837600	31°52′	85°58′	4851	Near a small lake next to the main road	2018/7/14
Plant 21	ZW-GZ-4	*Elymus atratus*	8029147	31°52′	85°58′	4851	Near a small lake next to the main road	2018/7/14
Plant 7	ZW-LJHB-1	*Stipa* sp.	2985667	31°31′	89°6′	4733	Next to the southern lakeside of the Siling Co	2018/7/10
Plant 8	ZW-LJHB-2	*Stipa* sp.	8316351	31°31′	89°6′	4733	Next to the southern lakeside of the Siling Co	2018/7/10
Plant 9	ZW-LJHB-3	*Scirpus pumilus*	2581179	31°31′	89°6′	4733	Next to the southern lakeside of the Siling Co	2018/7/10
Plant 10	ZW-LJHB-5	*Carex moorcroftii*	1487365	31°31′	89°6′	4733	Next to the southern lakeside of the Siling Co	2018/7/10
Plant 11	ZW-LJHB-6	*Carex setosa*	1862162	31°31′	89°6′	4733	Next to the southern lakeside of the Siling Co	2018/7/10
Plant 1	ZW-LJSP-1	*Carex moorcroftii*	2203286	31°30′	89°12′	4800	On a terrace to the south of the Siling Co, with a brook nearby, far from the lakeside	2018/7/9
Plant 2	ZW-LJSP-2	*Stipa* sp.	5477550	31°30′	89°12′	4800	On a terrace to the south of the Siling Co, with a brook nearby, far from the lakeside	2018/7/9
Plant 3	ZW-LJSP-3	*Stipa* sp.	3014375	31°30′	89°12′	4800	On a terrace to the south of the Siling Co, with a brook nearby, far from the lakeside	2018/7/9
Plant 4	ZW-LJT01-1	*Kobresia littledalei*	6568467	31°30′	89°12′	4796	On a terrace to the south of the Siling Co, with a brook nearby, far from the lakeside	2018/7/9
Plant 5	ZW-LJT01-2	*Stipa* sp.	22542667	31°30′	89°12′	4796	On a terrace to the south of the Siling Co, with a brook nearby, far from the lakeside	2018/7/9
Plant 6	ZW-LJT01-8	*Carex moorcroftii*	8502553	31°30′	89°12′	4796	On a terrace to the south of the Siling Co, with a brook nearby, far from the lakeside	2018/7/9
Plant 51	ZW-LSQ-1	*Pennisetum alopecuroides*	2898245	29°19′	90°40′	3590	At the Lhasa river valley, near the main road	2018/8/3
Plant 52	ZW-LSQ-3	*Eragrostis nigra*	16370640	29°19′	90°40′	3590	At the Lhasa river valley, near the main road	2018/8/3
Plant 22	ZW-MLDP-1	*Kobresia deasyi*	2902596	32°13′	81°14′	4587	From the bank of Shiquan River to the nearby mountain slope	2018/7/15
Plant 23	ZW-MLDP-4	*Elymus* sp.	620223	32°12′	81°14′	4590	From the bank of Shiquan River to the nearby mountain slope	2018/7/15
Plant 46	ZW-MLDPSD-1	*Poa crymophila*	5836235	32°13′	81°14′	4662	On a mountain top near the Shiquan River	2018/7/24
Plant 24	ZW-SG-3	*Scirpus and Carex*	2340833	32°23′	80°15′	4743	At a river bank not far from the main road	2018/7/15
Plant 32	ZW-SQHZ-1	*Elymus sibiricus*	4770000	32°24′	80°1′	4321	Open area near the Shiquanhe Town	2018/7/18
Plant 33	ZW-SQHZ-2	*Elymus* sp.	6112667	32°24′	80°1′	4321	Open area near the Shiquanhe Town	2018/7/18
Plant 34	ZW-SQHZ-3	*Poa* sp.	9304444	32°24′	80°1′	4321	Open area near the Shiquanhe Town	2018/7/18
Plant 12	ZW-WL-1	*Stipa* sp.	37206000	31°47′	87°29′	4540	Inside a fence that used to prevent overgrazing, much taller than the outside ones	2018/7/12
Plant 13	ZW-WL-2	*Stipa* sp.	14139478	31°47′	87°29′	4540	Outside the fence, much smaller than the inside ones	2018/7/12
Plant 50	ZW-YHX-1	*Pennisetum alopecuroides*	33452138	32°31′	82°28′	4895	Open area near the main road	2018/8/2
Plant 14	ZW-YPS-1	*Stipa purpurea*	3796168	31°10′	86°48′	4922	In the vally next to the Tangra Yumco	2018/7/13
Plant 15	ZW-YPS-2	*Stipa* sp.	4056210	31°10′	86°48′	4922	In the vally next to the Tangra Yumco	2018/7/13
Plant 16	ZW-YPS-3	*Poa* sp.	3093832	31°10′	86°48′	4922	In the vally next to the Tangra Yumco	2018/7/13
Plant 17	ZW-YPS-4	*Salix bangongensis*	2	31°10′	86°48′	4922	In the vally next to the Tangra Yumco	2018/7/13
Plant 35	ZW-ZD-1	*Poa* sp.	4169333	31°29′	79°48′	3708	In Zanda county, near a brook	2018/7/20
Plant 36	ZW-ZD-10	*Populus alba*	425211	31°29′	79°48′	3708	In Zanda county, roadside	2018/7/20
Plant 37	ZW-ZD-2	*Carex crebra*	2907985	31°29′	79°48′	3708	In Zanda county, near a brook	2018/7/20
Plant 38	ZW-ZD-4	*Poa annua*	776259	31°29′	79°48′	3708	In Zanda county, near a brook	2018/7/20
Plant 39	ZW-ZD-7	*Elymus* sp.	1076749	31°29′	79°48′	3708	In Zanda county, near a brook	2018/7/20
Plant 40	ZW-ZD-8	*Achnatherum inebrians*	1844833	31°29′	79°48′	3708	In Zanda county, near a brook	2018/7/20
Plant 41	ZW-ZD-9	*Phragmites australis*	3376100	31°29′	79°48′	3708	In Zanda county, near a brook	2018/7/20
Plant 42	ZW-ZDH-2	*Pennisetum alopecuroides*	20597474	31°30′	80°1′	4608	Open area near the main road	2018/7/20
Plant 43	ZW-ZDH-3	*Stipa* sp.	5811101	31°30′	80°1′	4608	Open area near the main road	2018/7/20
Plant 29	ZW-ZDQ-1	*Poa alpina*	1987320	31°8′	80°44′	4340	Open area between the main road and the bank of Xiangquan River	2018/7/17
Plant 30	ZW-ZDQ-2	*Pennisetum alopecuroides*	4669889	31°8′	80°44′	4340	Open area between the main road and the bank of Xiangquan River	2018/7/17
Plant 31	ZW-ZDQ-3	*Poa* sp.	4724571	31°8′	80°44′	4340	Open area between the main road and the bank of Xiangquan River	2018/7/17

*Except Hordeum vulgare var. nudum, the aerial parts of other specimens have not been separated for phytolith extraction.*

## Results

Phytoliths are found in three families: Poaceae, Cyperaceae, and Salicaceae. The highest production is 37 million grains/g in a *Stipa* sp. sample, while the lowest is two grains/g in *S. bangongensis*, and all other samples are higher than 0.4 million grains/g (Table 1). In Poaceae plants, phytolith production range from 0.6 million to 37.2 million grains/g, with an average of 7.2 million grains/g; in Cyperaceae plants, phytolith production range from 1.4 million to 8.5 million grains/g, with an average of 3.3 million grains/g; in Salicaceae plants, *S. bangongensis* barely produces phytoliths while *Populus alba* produces 0.4 million grains/g. Phytolith production is lowest in Salicaceae and highest in Poaceae; the production of Poaceae plants is twice higher than that of Cyperaceae with a larger SD (8258549.7 for Poaceae and 2316185.8 for Cyperaceae).

There are 22 morphotypes of phytoliths observed in the studied species, including diagnostic morphotypes produced in specific subfamilies and common morphotypes found in different families.

**FIGURE 2:**
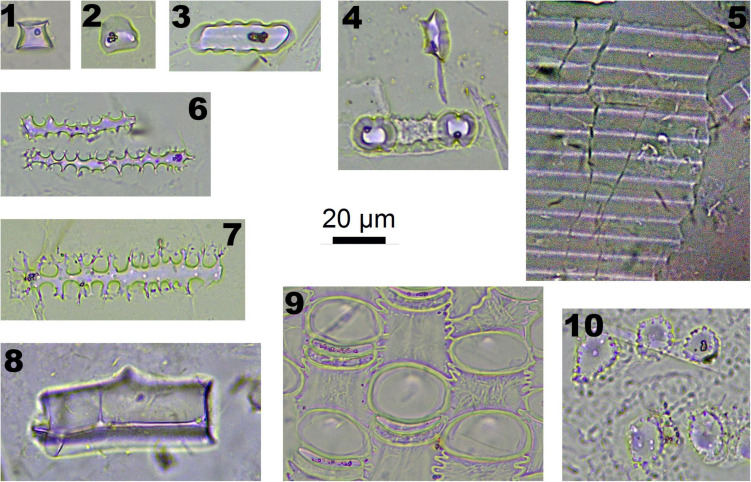
Phytoliths morphotypes in Pooideae plants. 1. RONDEL CONICAL; 2. RONDEL CARINATE; 3. CRENATE SINUATE; 4. BILOBATE SADDLE; 5. SILICIFIED EPIDERMIS; 6 and 7. ELONGATE DENDRITIC; 8. BLOCKY; 9. PAPILLATE CIRCULAR; and 10. PAPILLATE RADIATE.

**FIGURE 3:**
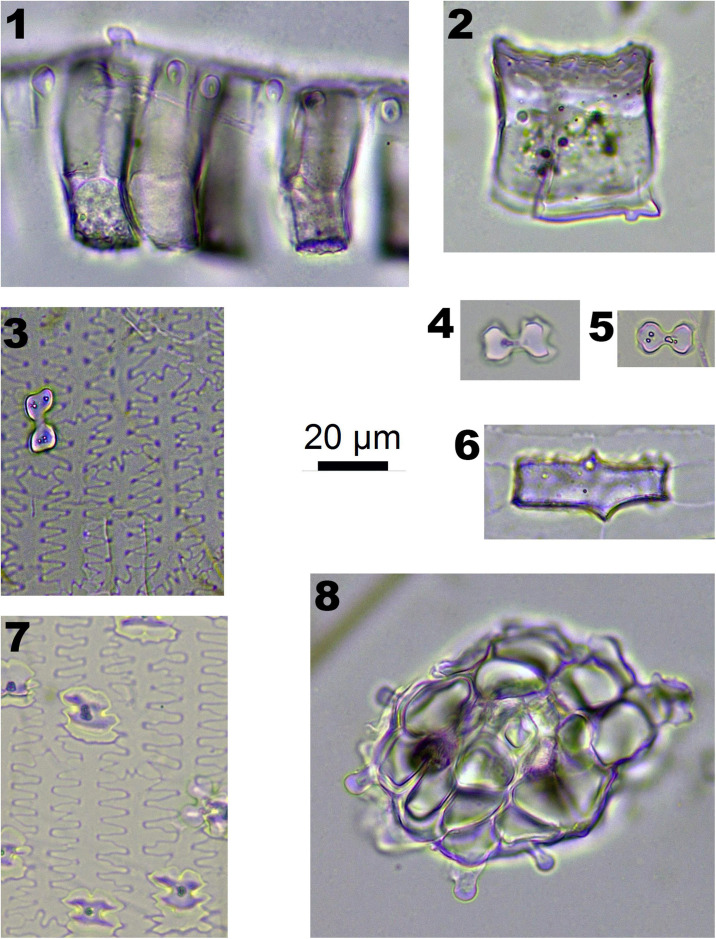
Phytoliths morphotypes in Panicoideae plants. 1 and 2. Bottom and side view of BULLIFORM; 3 and 7. INTERDIGITATING; 4 and 5. BILOBATE; 6. BLOCKY; and 8. SPHEROIDAL FAVOSE.

**FIGURE 4:**
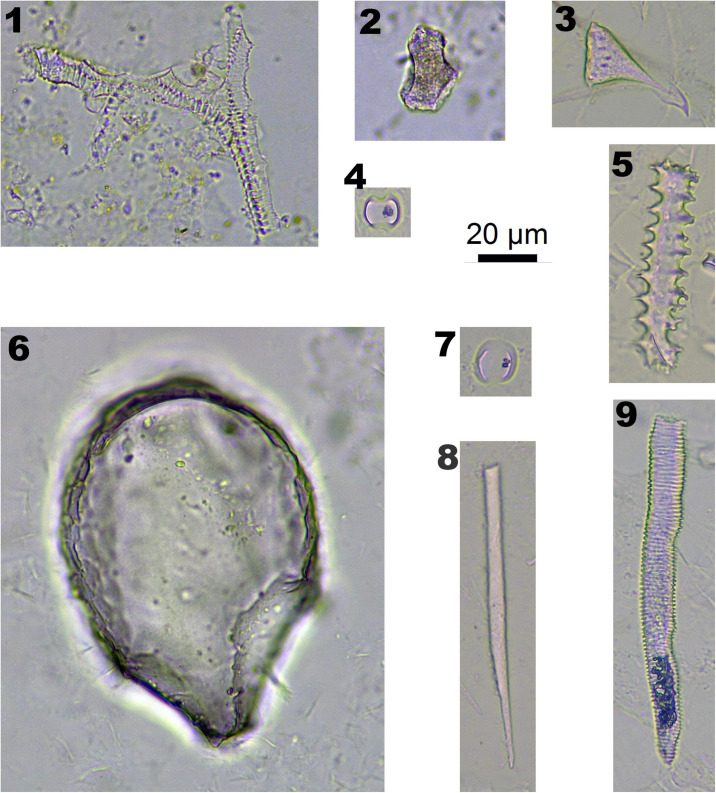
Phytoliths morphotypes in Arundinoideae, Chloridoideae, and Salicaceae. 1. TRACHEARY, in *Populus alba*; 2. BLOCKY, in *Populus alba*; 3. ACUTE BULBOSUS, in Poaceae and Cyperaceae plants; 4. SADDLE, in *Eragrostis nigra*; 5. ELONGATE ECHINATE, in Poaceae and Cyperaceae plants; 6. BULLIFORM FLABELLATE, in *Phragmites australis*; 7. SADDLE, in *Phragmites australis*; 8. ELONGATE ENTIRE, in Poaceae and Cyperaceae plants; and 9. TRACHEARY ELONGATE, in Poaceae and Cyperaceae plants.

**FIGURE 5:**
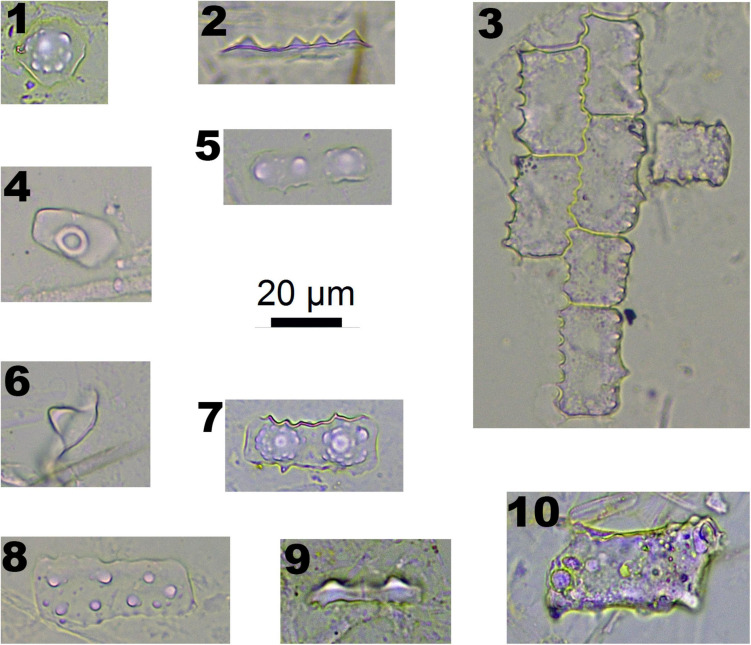
Phytoliths morphotypes in Cyperaceae plants. 1. PAPILLATE SINGULAR; 2,5, and 8. PAPILLATE MULTIPLE; 3. RECTANGULAR DENTATE; 4 and 6. PAPILLATE SINGULAR; 7 and 9. PAPILLATE BINATE; and 10. BLOCKY.

**FIGURE 6:**
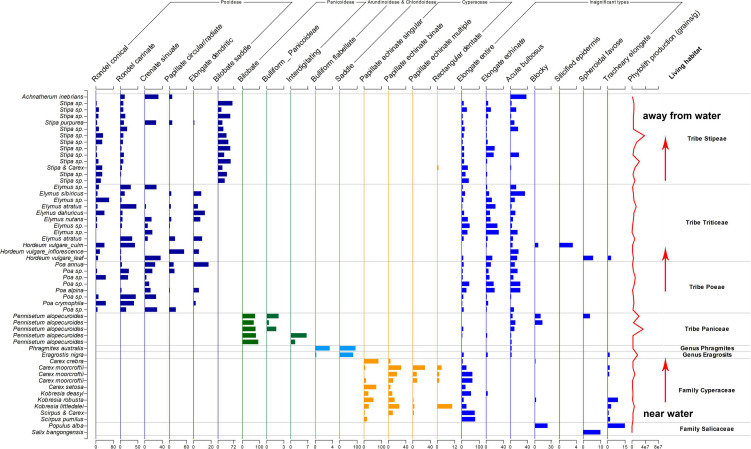
Phytoliths assemblages in studied species.

In Pooideae plants, RONDEL CONICAL (Figure 2-1), RONDEL CARINATE (Figure 2-2), CRENATE SINUATE (Figure 2-3), BILOBATE SADDLE (Figure 2-4), ELONGATE DENDRITIC (Figure 2-6,7) and PAPILLATE CIRCULAR/RADIATE (Figure 2-9,10) are diagnostic phytolith types; BLOCKY (Figure 2-8) and SILICIFIED EPIDERMIS (Figure 2-5) are insignificant types. ELONGATE DENDRITIC and PAPILLATE CIRCULAR/RADIATE originated from the inflorescence, SILICIFIED EPIDERMIS originated from the straw, and other types originated from leaves. BILOBATE SADDLE (Figure 2-4) from Tribe Stipae has a dumbbell-shaped base with one bracket-shaped ridge on each lobe; the two bracket-shaped ridges on each lobe together form the saddle top. On the other hand, the typical BILOBATE (Figure 3-4,5) from Panicoideae has a dumbbell-shaped base with two bracket-shaped ridges on each lobe. The morphological difference: one or two bracket-shaped ridges on top of the dumbbell base could be the discriminate criteria for distinguishing BILOBATE SADDLE from Tribe Stipae with BILOBATE from Panicoideae.

In Panicoideae plants (one species, *Pennisetum alopecuroides*), BILOBATE (Figure 3-4,5) and INTERDIGITATING (Figure 3-3,7) are diagnostic phytolith types. BULLIFORM (Figure 3-1,2), BLOCKY (Figure 3-1,2) and SPHEROIDAL FAVOSE (Figure 3-8) are insignificant types. BULLIFORM was found in three specimens, which originated from leaves. INTERDIGITATING was found in two specimens, which originated from the inflorescence.

In Arundinoideae (one species, *Phragmites australis*) and Chloridoideae (one species, *Eragrostis nigra*), *Bulliform flabellate* (Figure 4-6) and *Saddle* (Figure 4-4,7) are diagnostic types. Although these two specimens were collected at the lower elevation basin area (around 3,700 m), they were not observed in the high elevation area (over 4,000 m).

In Cyperaceae, PAPILLATE phytolith is the dominant and diagnostic type. PAPILLATE phytoliths have three major morphotypes, PAPILLATE SINGULAR (Figure 5-1,4,6) with one conical papilla, PAPILLATE BINATE (Figure 5-7,9) with two conical papillae and PAPILLATE MULTIPLE (Figure 5-2,5,8) with multiple conical papillae. Another potential diagnostic type is RECTANGULAR DENTATE (Figure 5-3) which originated from the inflorescence. It has a rectangular shape with dentate long sides and smooth short sides.

In Salicaceae, TRACHEARY (Figure 4-1) and BLOCKY (Figure 4-2) were observed in *P. alba*, and only one SPHEROIDAL FAVOS phytolith was observed in *S. bangongensis*. However, these types of phytoliths could also be found in other plants.

Some phytolith types could be observed in many species, which are defined as insignificant types, including ACUTE BULBOSUS (Figure 4-3), ELONGATE ECHINATE (Figure 4-5), ELONGATE ENTIRE (Figure 4-8), and TRACHAERY ELONGATE (Figure 4-9). These phytolith types are common in both Poaceae and Cyperaceae plants.

The assemblages of phytoliths types showed significant differences on the family level, tribe level or genus level, as shown in Figure 6. It is possible to distinguish different taxon using phytoliths assemblages on the TP.

## Discussion

In Tibetan Plateau, overgrazing is the primary biotic stress during the growing season, and grassland degradation has become a major threat to the sustainable development of livestock raising (Niu et al., 2019; Zhan, 2020). As a result, most samples collected in this study have been eaten by herbivores, the aerial parts are no higher than 20 cm. Only one specimen (*Stipa* sp.) was collected inside a closed fence, which grew 50 cm higher, and the phytolith production is the highest of all samples (37 million grains per gram of dry weight). While the specimen collected outside the fence grew only no higher than 10 cm, and the phytolith production was much lower (14 million grains per gram of dry weight). It has been known that phytoliths deposit while plants grow (Ma and Yamaji, 2006) and increase with the evapotranspiration (Madella et al., 2009): inside the closed fence, plants could grow better and have higher evapotranspiration; thus, they accumulate more phytoliths. In recent years, phytoliths have been considered a long term carbon sink (Carter, 2009; Song et al., 2022). However, in Tibet, overgrazing influenced the biomass of plants and decreased the production of phytoliths, which might eventually influence the sustainable development of grassland and carbon sink in these regions.

On the northern Tibetan Plateau, most plants grow during the growing season (Che et al., 2014). Thus, the temperature and precipitation are the same during the phytolith accumulation in the sampling sites (Hu et al., 2022). However, humidity showed to bea more critical factor in the growth of plants (Fu and Shen, 2016). During the exploration and sampling, we found that Cyperaceae plants only grew in or near the open water and formed sedge communities; away from the open water, Poaceae plants became dominant species and formed grass communities. Within the Poaceae plants, *P. australis* and *E. nigra* grew near water, *P. alopecuroides* grew near, or not far from open water, *Stipa* sp. grew better in drier areas than *Poa* sp. Thus, the phytoliths producers in this study showed that the hydrological conditions influenced their distribution; in other words, the vegetation changed along the hydrological gradient. In many cases, phytolith types have been considered to represent particular climate and environment types (Fredlund and Tieszen, 1997; Boyd, 2005; Gu et al., 2007; Li et al., 2017; Zuo et al., 2020). In China, RONDEL and CRENATE phytoliths from Pooideae plants indicated a cooler climate, BILOBATE phytoliths from Panicoideae plants indicated a warmer climate and PAPILLATE phytoliths from Cyperaceae plants were found to indicate a wetter environment (Wang and Lu, 1993). In contrast, the phytoliths types in Tibet might reflect the hydrological condition: PAPILLATE, BULLIFORM FLABELLATE, and SADDLE might indicate a strong humid environment, BILOBATE might indicate a less strong humid environment, RONDEL and CRENATE might indicate a medium humid environment, and BILOBATE SADDLE might indicate a relative drier environment. Such differences in the relationship between phytoliths types and environmental factors would lead to the different explanations of phytoliths combination in soil profiles on Tibetan Plateau, which also emphasizes the importance of regional study before the implication of phytolith analysis.

## Conclusion

This study explored the phytolith production and types in modern plants on the Tibetan Plateau. The results showed that the major phytolith producers are Poaceae and Cyperaceae plants, and we have found that BILOBATE SADDLE might be the new diagnostic type for the discrimination of Tribe Stipeae. Furthermore, phytoliths morphotypes and aseemblages on the TP respond to the environmental hydrological gradients rather than temperature compare with other regions in China: PAPILLATE, BULLIFORM FLABELLATE, SADDLE, BILOBATE represented a more humid environment, RONDEL, CRENATE, and BILOBATE SADDLE represented a relative dryer environment. These new data on modern phytoliths morphotypes and assemblages, as well as their response to the hydrological conditions could help future studies on the identification of phytoliths origins and the reconstruction of paleohydrological conditions.

## Data Availability Statement

The original contributions presented in this study are included in the article/supplementary material, further inquiries can be directed to the corresponding author/s.

## Author Contributions

YG and XZ designed the research. YG, YJ, and XZ collected the samples. YG performed the experiment, and carried out the image process and data analysis. All authors were involved in the writing and discussion of the manuscript, read and approved the final manuscript.

## Conflict of Interest

The authors declare that the research was conducted in the absence of any commercial or financial relationships that could be construed as a potential conflict of interest.

## Publisher’s Note

All claims expressed in this article are solely those of the authors and do not necessarily represent those of their affiliated organizations, or those of the publisher, the editors and the reviewers. Any product that may be evaluated in this article, or claim that may be made by its manufacturer, is not guaranteed or endorsed by the publisher.
